# Obesity Intervention Improves Outcomes in Socially Isolated Older Adults: Progress Despite a Pandemic

**DOI:** 10.1093/geroni/igaa057.3488

**Published:** 2020-12-16

**Authors:** Kathryn Porter Starr, Michael Borack, Marshall Miller, Angela Fruik, Jamie Rincker, Jessica Wallis, Amir Hakim, Connie Bales

**Affiliations:** 1 Duke University School of Medicine, Durham, North Carolina, United States; 2 Duke University, Durham, North Carolina, United States; 3 Duke University, Efland, North Carolina, United States

## Abstract

The extended social isolation necessitated by the COVID-19 pandemic will likely have a prolonged negative impact on the health of community-dwelling older adults. We studied the potential to counteract these negative effects, examining the before and after measurements of participants in two obesity intervention studies that were converted from in-person to remote delivery, due to COVID-19. The Veterans Achieving Weight Loss and Optimizing Resilience-Using Protein study (VALOR-UP; n=9) and the Egg-Supplemented Pre-Diabetes Intervention Trial (EGGSPDITE; n=6) enrolled obese (BMI = 34.2±3.8 kg/m2) older adults (age = 71.5±4.5 yrs; 80% male; 47% black) with prediabetes (fasting blood glucose 100-125 mg/dL and/or HbA1c of 5.7-6.4%). Participants followed a hypocaloric diet, attended weekly support groups, and weighed themselves weekly; VALOR-UP participants also attended a weekly exercise class delivered remotely. Outcomes were assessed at baseline (pre-COVID) and the end of a 4-month period coinciding with stay-at-home orders. Between baseline and 4 months, calorie intakes (3-day diet record) decreased by 402.1±529.3 kcal/day (p<0.05) and body weight by 6% (6.1±3.4 kg; p<0.0001). Physical function (Short Physical Performance Battery) improved by 1.1±1.4 units (p<0.01) and Short Form Health Survey (SF-36) physical and mental composite quality of life remained stable (p=0.63 and p=0.48, respectively). Thus, despite COVID-19 circumstances, most participants were able to benefit from an intervention to improve function and reduce obesity. The somewhat surprising findings for this small cohort offer promise not only for future lifestyle interventions during COVID-19 isolation but also for other isolated populations, including home-bound older adults.

